# Identifying Subgroups Among Current Smokers Enrolled in the Smoking Cessation Clinic Program: A Latent Class Analysis Approach

**DOI:** 10.3390/healthcare14101275

**Published:** 2026-05-08

**Authors:** Mi Sook Jung, Ah Rim Lee, Sok Goo Lee, Jeongeun Hwang, Nondumiso Satiso Dlamini

**Affiliations:** 1 College of Nursing, Chungnam National University, Daejeon 35015, Republic of Korea; 2 Department of Preventive Medicine, College of Medicine, Chungnam National University, Daejeon 35015, Republic of Korea; 3 Faculty of Health Sciences, University of the Witwatersrand, Johannesburg 2050, South Africa

**Keywords:** community health centers, health behavior, latent class analysis, smoking cessation

## Abstract

Background: Smoking cessation outcomes vary widely across individuals, yet clinic-based services often rely on uniform intervention strategies. Understanding heterogeneity in smoking behaviors, co-occurring health behaviors, and cessation-related psychological profiles may help refine community-based cessation services. This study aimed to identify distinct subgroups of smokers enrolled in public health center-based smoking cessation clinics and to examine differences in cessation outcomes and related characteristics across subgroups. Methods: Data from 21,105 newly enrolled adults attending 16 public health center-based smoking cessation clinics in C Province were drawn from the Smoking Cessation Service Integrated Information System of the National Tobacco Control Center. Latent class analysis was conducted using five indicators: time to first cigarette, cigarettes per day, prior quit attempts, alcohol use, and physical activity. Associations between latent class membership, covariates, and distal outcomes were examined using a three-step approach. Results: Four distinct latent classes were identified: *Sedentary heavy smokers* (46.8%), *Active social heavy smokers* (34.6%), *Inactive-nicotine addictive-light smokers* (13.6%), and *Active lifestyle light smokers* (5.0%). The latent classes showed different socioeconomic, smoking-related, and cessation-related confidence. Six-month cessation successes differed across classes, with higher abstinence among light-smoking classes and among *Sedentary Heavy Smokers* relative to *Active Social Heavy Smokers*. Conclusion: These findings highlight the value of person-centered profiling for informing more precise, context-sensitive cessation strategies and support the integration of behavioral and environmental considerations into community-based tobacco control programs.

## 1. Introduction

Tobacco smoking remains a critical public health challenge and a leading cause of preventable mortality worldwide, contributing substantially to chronic conditions such as ischemic heart disease, chronic obstructive pulmonary disease, and lung cancer [1]. Despite sustained global tobacco control efforts, declines in smoking prevalence have not consistently translated into proportional reductions in smoking-attributable mortality [2]. In South Korea, a marked decline in smoking prevalence ranging from 35.1% in 1998 to 19.6% in 2023 has not been accompanied by a comparable reduction in smoking-related mortality [3,4]. This pattern suggests a persistent disconnection between declining smoking prevalence and sustained smoking-attributable mortality, underscoring the continued need for effective strategies to prevent smoking initiation and promote cessation.

A nationwide Smoking Cessation Assistance Program was established in 2005 under the National Health Promotion Act [5]. Public health centers in regional areas function as the primary access points, delivering a six-month, cost-free, community-based cessation program through trained providers, including registered nurses. Program participation was initially high but has since declined, a trajectory mirrored by reduction in cessation success. Enrollment fell from more than 574,000 participants in 2015 to 211,860 in 2023, while cessation success rates decreased from over 40% in 2016 to 32.6% in 2023 [6]. Such a downward trajectory underscores the case for refining clinic-based cessation services to sustain engagement and strengthen long-term outcomes [7].

Previous research consistently shows that smoking cessation success is strongly influenced by the degree of nicotine dependence, as reflected in indicators such as smoking within 30 min of waking, higher daily cigarette consumption, and a limited history of prior quit attempts [8,9]. Cessation outcomes are also shaped by co-occurring health behaviors [10,11]. Alcohol use, in particular, has been shown to undermine quit attempts by increasing exposure to situational cues and intensifying craving and relapse vulnerability [12,13]. In contrast, evidence regarding the role of physical activity is less consistent. While physical activity may alleviate stress and withdrawal symptoms, its effects on cessation appear to vary by type and intensity, and adjunctive exercise interventions have not demonstrated uniform benefits for cessation outcomes [14,15].

These mixed findings highlight the importance of examining smoking cessation within the broader context of multiple co-occurring health behaviors rather than in isolation. Among adults aged 50 years and older, smoking frequently occurs alongside alcohol use and low levels of physical inactivity, a combination that can complicate efforts to quit [10]. Such clustering of risk behaviors has been associated with lower readiness to quit, even among individuals who report motivation or confidence to do so [16]. Similar patterns have been observed in Korea. Smokers often engage in multiple unhealthy behaviors, and relatively few exhibit consistently health-promoting profiles [11]. Collectively, this evidence suggests that smoking cessation may be better understood when concurrent health behaviors are considered alongside smoking-related characteristics [17,18].

Latent class analysis (LCA) is a person-centered approach that identifies unobserved subgroups defined by distinct patterns of responses across observed indicators and assigns individuals to latent classes probabilistically [19]. Unlike variable-centered methods, which estimate average associations under assumptions of population homogeneity, LCA is useful for identifying heterogeneous behavioral profiles. This approach is particularly relevant in smoking research, where smoking-related and co-occurring health behaviors often cluster in complex ways across individuals. Accordingly, the present study applied LCA to smokers enrolled in public health center-based cessation clinics in South Korea to identify behavioral subgroups and to examine subgroup differences in sociodemographic characteristics, cessation-related factors, and cessation success. Rather than examining smoking-related, behavioral, and psychological correlates separately, the present study applied a person-centered approach to identify their combined profiles within a large real-world clinical population. The primary contribution of this study is the identification of behaviorally and psychologically distinct smoker subgroups within routine public health center-based cessation services, based on a clinically relevant combination of smoking-related and co-occurring health behavior indicators.

## 2. Methods

### 2.1. Study Sample

This study employed a secondary analysis of data drawn from the Smoking Cessation Service Integrated Information System (SCSIIS) of the National Tobacco Control Center in South Korea. The SCSIIS compiles administrative and clinical records for individuals enrolled in community-based smoking cessation clinics and tracks their cessation progress over a six-month period. Under the standard protocol of the national smoking cessation support program, participants who self-report abstinence at six months were invited to undergo biochemical verification to confirm smoking status.

The study was reviewed and approved by an appropriate institutional review board. The National Tobacco Control Center authorized the use of the SCSIIS data. The dataset was de-identified, no direct contact with participants occurred, and no personally identifiable information was accessed. Written consent for program participation and data collection was obtained from all participants at clinic enrollment.

**Figure 1:**
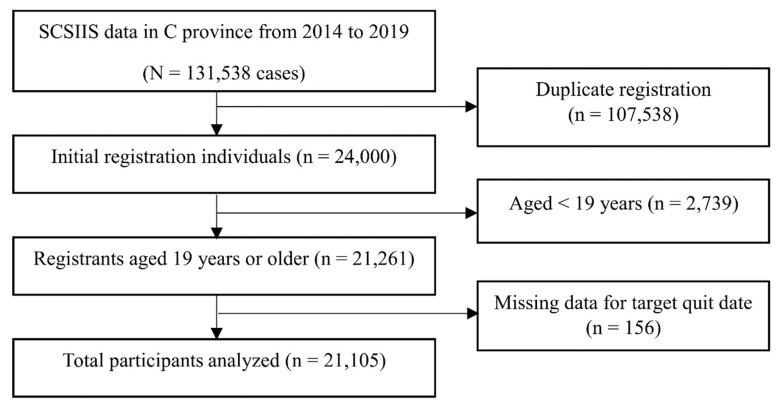
Participant Select Process.

The study included adult new registrants in the smoking cessation clinic program in C Province between 2014 and 2019. Registered smokers were broadly defined as individuals currently using tobacco and/or non-pharmaceutical nicotine delivery products who enrolled in the program, rather than being limited to combustible cigarette smokers only. The dataset included both re-registrants (i.e., individuals who had previously enrolled but did not achieve cessation) and first-time registrants. Given evidence that cessation mechanisms differ substantially between these groups [20], the present study was restricted to new registrants. Individuals were excluded if they were younger than 19 years or had missing outcome data. The original dataset comprised 131,538 records. After excluding duplicate records corresponding to re-registrants (*n* = 107,538), individuals younger than 19 years (*n* = 2739), and cases with missing outcome data (*n* = 156), the final analytic sample consisted of 21,105 adults (Figure 1).

### 2.2. Measures

The study variables were derived from enrollment and follow-up assessments recorded in the SCSIIS [6]. Study variables were organized as latent class indicators, covariates, and cessation outcomes.

**Latent Class Indicators.** Five variables were selected as latent class indicators based on prior evidence linking these constructs to smoking cessation outcomes [8,10,15,21], rather than data-driven selection procedures. To enhance interpretability, maintain consistency with clinically meaningful thresholds commonly used in smoking research, and ensure model parsimony, indicators were specified as binary variables [22]. Dichotomization was also considered appropriate to reduce potential sparse cell issues in latent class model estimation [23,24]. Although dichotomization may involve some loss of information, and reduce variability in the indicators, potentially influencing latent class formation, it was considered appropriate to facilitate clinically interpretable subgroup identification and stable class estimation. Nicotine dependence was indexed using two behavioral markers derived from the Fagerström Test for Nicotine Dependence (FTND): time to first cigarettes after waking (≤30 vs. >30 min) and cigarettes per day, dichotomized as >10 versus ≤10. Cessation history was assessed using a past-year quit attempt (yes/no). Co-occurring health behaviors included alcohol use during the past year (yes/no) and regular physical activity (yes/no), operationalized as participation in moderate-intensity activity for 10 min or more per day. Alcohol use and physical activity were originally collected as dichotomous variables in the source dataset.

**Covariates.** Demographic covariates included sex, age, and education level. Smoking-related covariates included smoking duration, calculated as age at registration minus age at initiation, baseline expired-air carbon monoxide (CO) concentration measured with a portable CO monitor, and duration of the longest quit attempt during the past year, recorded in days, with participants reporting no quit attempt coded as 0 [6]. In addition, a cessation-related psychological profile was assessed at enrollment using a single-item 10-point scale measuring motivation and readiness to quit. Confidence to quit smoking was assessed repeatedly at each clinic visit using a single-item rating.

**Smoking Cessation Success.** Six-month smoking status was assessed at the scheduled follow-up visit by self-report. Participants reporting abstinence were invited to undergo biochemical verification in accordance with national program guidelines using expired-air carbon monoxide (CO) testing, urine cotinine testing, or both. Expired-air CO concentration was measured during routine clinic visits using the standard procedure applied in the smoking cessation clinic program. Urine cotinine testing was conducted using standard procedures within the smoking cessation clinic program. For participants receiving nicotine replacement therapy, abstinence was verified using expired-air CO testing only [6]. Biochemically verified abstinence was defined as expired-air CO level <10 ppm and/or a negative urine cotinine result. No special preparation was required for expired-air CO measurement. Participants without biochemical verification were classified as non-abstinent using a conservative approach consistent with established recommendations in smoking cessation research [25].

### 2.3. Data Analysis

Descriptive statistics for all study variables were generated using SPSS 26.0 software (SPSS, Inc., Chicago, IL, USA). Latent class analysis (LCA) was performed in Mplus 8.7 software (Mplus, Los Angeles, CA, USA) to identify subgroups of smokers based on five indicator variables. Competing class solutions were compared using multiple fit indices, including the Akaike information criterion (AIC), Bayesian information criterion (BIC), sample-size-adjusted Bayesian information criterion (SABIC), entropy, Lo-Mendell-Rubin likelihood ratio test (LMRT), and bootstrapped likelihood ratio test (BLRT). The final model was selected based on overall model fit, parsimony, and substantive interpretability [26]. Associations between latent class membership and baseline covariates were examined using multinomial logistic regression with the R3STEP procedure to account for classification uncertainty. Differences in distal outcomes across classes were evaluated using the DCAT approach [27].

## 3. Results

### 3.1. Sample Characteristics

The mean age of participants was 48.63 years (SD = 17.87). Most participants were male (91.0%), and 73.5% had completed at least a high school education. At enrollment, 69.5% reported smoking within 30 min of waking. The mean number of cigarettes smoked per day was 19.00 (SD = 9.80), with 77.9% smoking more than 10 cigarettes per day (Table 1). Past-year alcohol use was reported by 46.4% of them, and 27.4% reported engaging in regular physical activity. Mean scores for motivation, readiness, and confidence to quit were 8.39 (SD = 1.86), 7.64 (SD = 2.11), and 7.29 (SD = 2.15), respectively.

### 3.2. Latent Class Enumeration and Profiles

**Figure 2:**
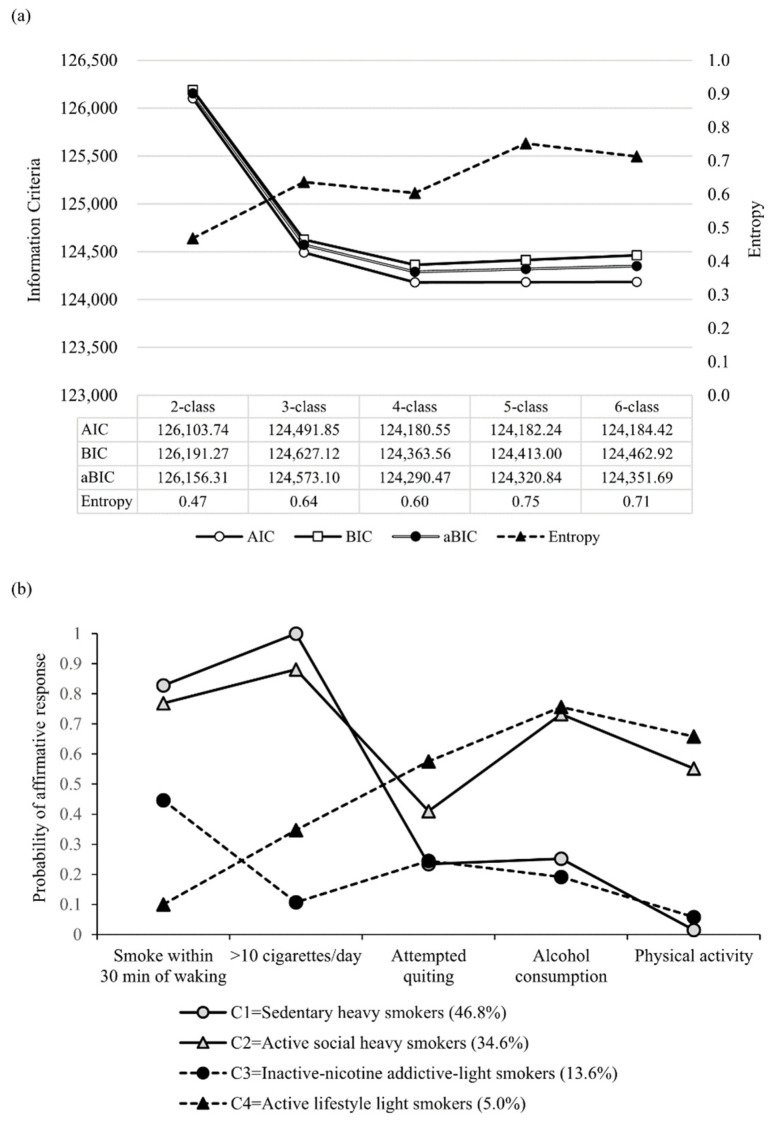
Model fit indices and item-response profiles for latent classes of smokers. (**a**) Information criteria and entropy across two- to six-class solutions. (**b**) Item-response probabilities for the four-class solution.

A series of latent class models specifying two through six classes was evaluated using multiple fit indices, including AIC, BIC, SABIC, entropy, LMRT, and BLRT. Model fit indices favored a four-class solution, indicated by the lowest BIC, and comparably lower AIC and SABIC, along with acceptable entropy. The LMRT and BLRT suggested that both four- and five-class solutions were statistically plausible, whereas the six-class solution was not supported. The five-class solution did not identify a substantively distinct new subgroup but instead split one of the profiles observed in the four-class model into two smaller subclasses with broadly similar patterns of low physical activity, low alcohol use, and limited quit-attempt history. Specifically, one subclass retained the characteristics of the original Sedentary Heavy Smoker profile, whereas the additional subclass appeared to represent a lower-smoking-intensity variant of this same general pattern. As a result, the five-class solution appeared to offer limited additional interpretive value and introduced a more fragmented class structure. It was not retained, as it did not provide a clearly differentiated additional class and reduced model parsimony. The four-class solution was selected because it provided clearer class separation, greater conceptual interpretability, and a more parsimonious representation of the data (Figure 2a). Based on item response probabilities, four distinct subgroups of smokers were identified. The largest subgroup, *Sedentary Heavy Smokers* (46.8%), was characterized by early smoking initiation and heavy cigarette consumption, accompanied by few prior quit attempts and minimal engagement in physical activity. The *Active Social Heavy Smokers* (34.6%) also exhibited heavy smoking but were more likely to report alcohol use, regular physical activity, and prior quit attempts. The *Inactive-Nicotine Addictive- Light Smokers* (13.6%) demonstrated moderate nicotine dependence with generally lower levels of cigarette consumption and limited co-occurring health behaviors. Finally, the smallest subgroup, *Active Lifestyle Light Smokers* (5.0%), showed the highest probabilities of prior quit attempts, alcohol use, and physical activity (Figure 2b).

### 3.3. Sociodemographic and Smoking-Related Characteristics Across Classes

Differences in sociodemographic and smoking-related characteristics across latent classes were examined using the R3STEP procedure, and the results are presented in Table 2. Relative to the *Active Lifestyle Light Smokers,* participants in all other classes were younger. Female smokers were disproportionately represented in the *Sedentary Heavy Smokers* and *Inactive-Nicotine Addictive-Light Smokers,* and these two classes also exhibited lower educational attainment, with the lowest levels observed among *Inactive-Nicotine Addictive-Light Smokers.* With respect to smoking-related characteristics, all three non-reference classes reported longer smoking histories and higher baseline expired-air CO levels than *Active Lifestyle Light Smokers*. *Sedentary Heavy Smokers* were also less likely to have sustained longer periods of prior abstinence. Psychological profiles at enrollment further also differed across the classes. Compared with *Active Lifestyle Light Smokers, Inactive-Nicotine Addictive-Light Smokers* reported relatively higher confidence to quit.

### 3.4. Distal Outcomes

Distal outcomes across latent classes were evaluated using the DCAT approach. Significant differences in smoking cessation success and confidence were observed across latent classes at the six-month follow-up (Table 3). The highest probabilities of six-month abstinence based on both self-reported and biochemically verified outcomes, were observed among *Inactive-Nicotine Addictive-Light Smokers,* followed by *Active Lifestyle Light Smokers.* Among the heavy-smoking classes, higher cessation success probabilities were observed among *Sedentary Heavy Smokers* than *Active Social Heavy Smokers.* Class differences were also observed in confidence at follow-up, with the highest levels reported among *Active Lifestyle Light Smokers*.

## 4. Discussion

This study identified four distinct subgroups of smokers enrolled in community-based smoking cessation clinics, revealing substantial heterogeneity in smoking behaviors, co-occurring health habits, and cessation-related psychological profiles. Notably, the identified classes differed not only in smoking intensity and nicotine dependence but also in patterns of alcohol use, physical activity, and readiness and confidence to quit. By applying a person-centered analytic approach, these findings provide a more integrated understanding of smoking-related behavioral, contextual, and psychological heterogeneity in real-world settings. This perspective highlights patterns that are not readily captured when these factors are examined separately.

Among heavy smokers, cessation outcomes differed between *Sedentary Heavy Smokers* and *Active Social Heavy Smokers* despite comparable indicators of nicotine dependence. These differences may reflect variation in co-occurring behavioral and contextual factors, such as alcohol use and social smoking environments, rather than nicotine dependence alone. In particular, alcohol use and socially reinforced smoking contexts may be associated with increased relapse risk beyond indicators of physiological dependence. Prior research has shown that drinking environments amplify cue-induced craving and undermine abstinence even after repeated quit attempts [12,28,29]. From this perspective, the observed patterns may reflect underlying behavioral and environmental characteristics represented within each class. These findings may help generate hypotheses regarding how behavioral and contextual profiles relate to cessation challenges and potential intervention targets, particularly those addressing alcohol-related cues and social smoking contexts, as well as combinations of pharmacotherapy and behavioral support [12,13].

Psychological patterns further differentiated the two light-smoking classes. The groups differed less in smoking intensity than in how confidence and readiness were expressed. *Active Lifestyle Light Smokers* tended to show a pattern characterized by higher readiness, whereas *Inactive-Nicotine Addictive-Light Smokers* reported relatively higher confidence despite lower readiness. This pattern may reflect a misalignment between perceived confidence and behavioral preparation, which could be relevant to sustained abstinence, particularly among individuals with residual nicotine dependence [30,31]. Overall, these findings suggest that differences in cessation outcomes across the two light-smoking classes may be related to variation in alignment between motivational beliefs and behavioral preparation, rather than smoking intensity alone.

Socioeconomic characteristics also differed across classes. *Inactive-Nicotine Addictive-Light Smokers* were more likely to be female and to have lower educational attainment. This pattern is broadly consistent with prior evidence showing that socioeconomic disadvantage is associated with lower readiness to quit and with the clustering of multiple health risk behaviors [32]. These findings suggest that cessation-related behaviors and psychological profiles may reflect not only individual characteristics but also broader social and structural conditions. In this context, community-based cessation clinics may represent relevant settings for considering multicomponent and equity-oriented smoking cessation support, particularly for smokers with co-occurring behavioral and socioeconomic vulnerabilities.

Differences in distal outcomes were observed across the latent classes. Overall, light-smoking classes had higher cessation success than heavy-smoking classes, which may be related to lower nicotine exposure and more favorable behavioral profiles [10,33,34]. Among heavy smokers, *Active Social Heavy Smokers* had lower cessation success than *Sedentary Heavy Smokers* despite similar levels of nicotine dependence. In addition, the discrepancy between self-reported and biochemically verified abstinence highlights the importance of objective outcome assessment in cessation research and practice [25]. Taken together, these findings suggest that the latent classes summarize meaningful patterns of co-occurring characteristics associated with cessation outcomes. However, the observed differences across classes may partly reflect underlying covariates, such as age, smoking duration, or socioeconomic conditions, rather than latent class membership itself.

This study has several strengths. First, it used a large, real-world sample drawn from routine public health center-based smoking cessation services, which enhances the practical relevance of the findings to community-based smoking cessation care. Second, the person-centered analytic approach allowed for the identification of heterogeneous behavioral and psychological profiles among smokers beyond average group-level effects. Third, the inclusion of biochemically verified cessation outcomes strengthened measurement validity and reduced reliance on self-reported abstinence alone.

Several limitations should also be acknowledged. First, latent classes were identified cross-sectionally, which limits causal interpretation of the observed associations between class membership and cessation outcomes. Second, some latent class indicators were operationalized as dichotomous variables. Although categorical indicators are commonly used in latent class analysis and support clinical interpretability, the use of five binary indicators may have reduced variability and influenced class separation depending on the selected cut points. In particular, alcohol use and physical activity were measured dichotomously and therefore may not have captured meaningful behavioral variation. In addition, the available dataset lacked more detailed behavioral and psychosocial variables, which may have limited more nuanced characterization of smoker subgroups. Third, several behavioral measures were based on self-report and may have been subject to recall or social desirability bias. In addition, classifying participants without biochemical verification as non-abstinent may have resulted in conservative estimates of cessation success. Finally, this study did not directly evaluate whether different cessation strategies were more effective for specific latent classes. The intervention-related implications of the identified class structure should be interpreted cautiously and regarded as hypothesis-generating rather than as evidence of differential treatment effects. Demonstrating the practical value of this categorization would require future studies that develop subgroup-specific treatment protocols and compare them with a standard one-size-fits-all approach. Future longitudinal studies are needed to examine the stability of latent class membership and its temporal relationship with cessation outcomes.

These findings provide a descriptive framework for understanding heterogeneity in smoking-related behavioral and psychological profiles in community-based settings. While this may inform hypothesis generation, its practical utility for guiding tailored smoking cessation interventions remains to be established. Demonstrating such utility would require future studies that develop subgroup-specific intervention protocols and directly compare their effectiveness with standard one-size-fits-all approaches using appropriate control groups. In this context, the identified profiles may serve as a basis for future investigation rather than as evidence to support differential treatment strategies.

## 5. Conclusions

This study delineated distinct patterns of smoking behavior, co-occurring health behaviors, and cessation-related psychological characteristics among smokers enrolled in community-based clinics. The observed variation in cessation outcomes across these subgroups highlights the importance of considering how these dimensions cluster within individuals in real-world clinical settings. These findings extend existing approaches by integrating behavioral and psychological dimensions within a person-centered framework. Although the present study does not evaluate intervention effectiveness, the identified profiles provide a basis for future research examining how such multidimensional patterns may be associated with variation in cessation challenges and outcomes.

## Figures and Tables

**Table 1:** Demographic characteristics (*n* = 21,105).

Variable	*n* (%) or M ± SD
Age (years)	48.63 ± 17.87
Sex, female	1906 (9.00)
Education level, ≥High school	11,478 (73.5)
Alcohol use, yes	9775 (46.4)
Physical activity, yes	5771 (27.4)
Smoking-related behaviors	
Smoked within 30 min of waking	14,660 (69.5)
Average number of cigarettes per day	19.00 ± 9.80
Cigarettes per day, >10 cigarettes	16,434 (77.9)
Smoking duration (years)	27.45 ± 16.86
Attempts to quit smoking within the past year	6931 (32.8)
Baseline expired-air carbon monoxide level (ppm)	9.86 ± 8.84
Duration of smoking abstinence (days)	63.82 ± 261.49
Smoking cessation psychological profile (at enrollment)	
Motivation to quit smoking	8.39 ± 1.86
Preparation to quit smoking	7.64 ± 2.11
Confidence in quitting smoking	7.29 ± 2.15

*Abbreviations:* M = Mean, SD = standard deviation.

**Table 2:** Estimated odds ratios (OR) of smoker class membership based on covariates.

Variable	Reference: C4		
	C1	C2	C3
	OR (95% CI)	OR (95% CI)	OR (95% CI)
Age (years)	0.95 (0.94–0.97) **	0.90 (0.94–0.98) **	0.93 (0.94–0.97) **
Sex, female	1.86 (1.23–2.82) *	0.92 (0.61–1.38)	3.77 (2.40–5.90) **
Education level, ≥High school	0.56 (0.41–0.77) **	1.02 (0.75–1.38)	0.34 (0.24–0.48) **
Smoking duration (years)	1.08 (1.06–1.10) **	1.06 (1.04–1.08) **	1.07 (1.05–1.09) **
Baseline expired-air carbon monoxide level (ppm)	1.16 (1.14–1.18) **	1.14 (1.12–1.16) **	1.05 (1.03–1.08) **
Duration of smoking abstinence (days)	0.33 (0.20–0.57) **	1.00 (1.00–1.00)	0.31 (0.08–1.18)
Motivation to quit smoking at enrollment	1.11 (1.03–1.19) *	1.11 (1.03–1.19) *	1.14 (1.05–1.24) *
Preparation to quit smoking at enrollment	0.79 (0.73–0.86) **	0.92 (0.84–1.00) *	0.70 (0.64–0.77) **
Confidence in quitting smoking at enrollment	0.99 (0.92–1.07)	0.89 (0.82–0.96) *	1.17 (1.07–1.28) **

*Abbreviations*: C1 = *Sedentary Heavy Smokers*, C2 = *Active Social Heavy Smokers*, C3 = *Inactive–Nicotine Additive–Light Smokers*, C4 = *Active Lifestyle Light Smokers*. * *p* < 0.05, ** *p* < 0.001.

**Table 3:** Distal outcome comparisons of six-month cessation successes and confidence in quitting across latent class memberships.

Outcome Variable	C1	C2	C3	C4	*p*
Six-month success, probability (SE)					
self-reported	0.459 (0.011)	0.428 (0.009)	0.552 (0.015)	0.534 (0.020)	<0.001
biochemically verified	0.111 (0.006)	0.084 (0.006)	0.163 (0.009)	0.142 (0.018)	<0.001
Confidence in quitting smoking, M (SD)	8.30 (0.03)	8.43 (0.04)	8.45 (0.05)	8.67 (0.09)	<0.001

*Abbreviations*: M = Mean, SD = standard deviation, SE = standard error, C1 = *Sedentary Heavy Smokers*, C2 = *Active Social Heavy Smokers*, C3 = *Inactive-Nicotine Additive-Light Smokers*, C4 = *Active Lifestyle Light Smokers*.

## Data Availability

The individual-level data from the SCSIIS are not publicly available because of data protection restrictions and can be accessed only with authorization from the National Tobacco Control Center.
